# The Effect of Submaximal Exercise Preceded by Single Whole-Body Cryotherapy on the Markers of Oxidative Stress and Inflammation in Blood of Volleyball Players

**DOI:** 10.1155/2013/409567

**Published:** 2013-12-31

**Authors:** Celestyna Mila-Kierzenkowska, Alicja Jurecka, Alina Woźniak, Michał Szpinda, Beata Augustyńska, Bartosz Woźniak

**Affiliations:** ^1^The Chair of Medical Biology, Collegium Medicum of Nicolaus Copernicus University, Karłowicza 24, 85-092 Bydgoszcz, Poland; ^2^Department of Orthopaedics and Musculoskeletal Traumatology, Collegium Medicum of Jagiellonian University, Os. Zlotej Jesieni 1, 31-826 Krakow, Poland; ^3^Department of Normal Anatomy, Collegium Medicum of Nicolaus Copernicus University, Karłowicza 24, 85-092 Bydgoszcz, Poland; ^4^The Chair and Department of Biochemistry, Collegium Medicum of Nicolaus Copernicus University, Karłowicza 24, 85-092 Bydgoszcz, Poland; ^5^Department of Neurosurgery, Stanisław Staszic Specialist Hospital, Rydygiera 1, 64-920 Piła, Poland

## Abstract

The aim of the study was to determine the effect of single whole-body cryotherapy (WBC) session applied prior to submaximal exercise on the activity of antioxidant enzymes, the concentration of lipid peroxidation products, total oxidative status, and the level of cytokines in blood of volleyball players. The study group consisted of 18 male professional volleyball players, who were subjected to extremely cold air (−130°C) prior to exercise performed on cycloergometer. Blood samples were taken five times: before WBC, after WBC procedure, after exercise preceded by cryotherapy (WBC exercise), and before and after exercise without WBC (control exercise). The activity of catalase statistically significantly increased after control exercise. Moreover, the activity of catalase and superoxide dismutase was lower after WBC exercise than after control exercise (*P* < 0.001). After WBC exercise, the level of IL-6 and IL-1*β* was also lower (*P* < 0.001) than after control exercise. The obtained results may suggest that cryotherapy prior to exercise may have some antioxidant and anti-inflammatory properties. The relations between the level of studied oxidative stress and inflammatory markers may testify to the contribution of reactive oxygen species in cytokines release into the blood system in response to exercise and WBC.

## 1. Introduction

The physical effort is a complex systemic process, which may have many health benefits including a lowered threat of all-cause mortality along with a reduced risk of respiratory and cardiovascular disease, cancer, diabetes, and many others [1, 2]. However, it is also clear that high training volume and insufficient recovery may induce muscle damage with subsequent inflammation indicated by soreness, swelling, and loss of muscle function [3].

In modern sport, the competitive athletes are often exposed to high-intensity exercise performed multiple times per week, which may lead to overreaching and overtraining and diminish competitive performance [4]. The short period of recovery available for the athletes requires applying the additional methods that prevent overtraining [5]. Hence, in recent years, the scientific interest in sport medicine has been increasing in recovery modalities. A modern method used to maximize the performance of the athletes and to prevent traumas seems to be stimulation cryotherapy (cryostimulation). Initially, it was predominantly used in treating several diseases; however, its beneficial effects in postexercise recovery make it more and more often applied in sportsmen [6, 7]. For professional sportsmen, the preferred form of stimulation by cryogenic temperatures is whole-body cryotherapy (WBC). Whole-body cryotherapy refers to a brief exposure in minimal clothing to very cold air (below −100°C) to the whole surface of the body for 2-3 minutes [8]. The physiological reactions of organism that occur after the whole-body cryotherapy include analgetic, antiswelling, immune and circulatory system reactions, and beneficial effect in lipids profile [9, 10]. Systemic cryotherapy also stimulates the hypothalamus-hypophysis hormone axis and results in altered concentration of hormones such as corticosteroids, beta-endorphins, or norepinephrine [11]. Moreover, WBC shows positive effects on the muscular enzymes like creatine kinase and lactate dehydrogenase, which facilitates recovery in the athletes [6]. However, some reports demonstrate that exposure to extremely low temperatures may induce the generation of reactive oxygen species (ROS) in organism [12]. The possible sources of ROS after cryotherapy are, for example, global tissue hyperemia as a result of vasospasm and then vasodilation [13], the reaction catalyzed by xanthine oxidase as a consequence of blood vessels reperfusion [14], and an intensification of *β*-oxidation of fatty acids in the hepatocyte peroxisomes [15]. On the other hand, in accordance to the theory of hormesis, the antioxidant action of WBC is also postulated [16].

Despite the muscle soreness, the prolonged and strenuous exercise is known to increase oxygen metabolism in skeletal muscles, which results in intense ROS production and thus may lead to oxidative stress [17]. During the past decades, our knowledge about the biological implications of exercise-induced oxidative stress has expanded rapidly. Indeed, it is now acknowledged that high levels of ROS released during intense exercise can damage cellular components; however, low levels of free radicals play multiple regulatory roles such as control of gene expression, modulation of skeletal muscle force production, and regulation of the inflammation [18]. The local muscle damage occurring after exercise, especially if it is strenuous and includes eccentric muscle contractions, results in the release of various substances, which initiate the formation of a local inflammatory response [19]. This leads to increased permeability of blood vessels, which allows the migration of phagocytic cells from the blood stream to the area of myocyte damage with concomitant release of the great number of ROS [20]. Acute inflammatory response is associated with increase of migrating neutrophils present in damaged muscle fibers. The crucial role in activation of neutrophils is played by proinflammatory cytokines (myokines), such as IL-1*β* and TNF-*α*. The changes in cytokines profile after physical exercise accompanied by damage to the muscle fibres were previously reported by some authors and the level of released cytokines depended on the intensity of exercise, its duration, and activation of specific muscle area [21–23].

The main aim of this study was to investigate whether the single WBC session applied before the exercise may affect the activity of antioxidant enzymes like superoxide dismutase (SOD), catalase (CAT), and glutathione peroxidase (GPx) as well as the concentration of lipid peroxidation products, total oxidative status (TOS), and the level of selected cytokines: interleukin-1*β* (IL-1*β*), interleukin-6 (IL-6), tumor necrosis factor *α* (TNF-*α*), and transforming growth factor *β*1 (TGF-*β*1) in blood of professional volleyball players. We chose these parameters because they are reliable indicators of acute performance deterioration and muscle damage and they may play important role in the response of organism to thermal stress. The literature data in the field of WBC mainly consider the long-term effect of stimulation by cryogenic temperatures and previous researchers demonstrated different protocols of this method, that is, 5 sessions during 7 days [6], elevated number (5 to 20) of WBC sessions [10], or 36 sessions for 12 weeks [11]. Moreover, most of the previous studies were performed on the athletes during their training cycles and WBC was applied after the exercise to accelerate the regeneration. Yet, the studies on the effect of only single WBC intervention prior to exercise on parameters of both oxidative and inflammatory responses are still lacking. We hypothesized that the magnitude of exercise-induced oxidative stress may be partly reduced by single session of whole-body cryotherapy applied prior to the submaximal exercise.

## 2. Materials and Methods

The study was attended by 18 professional male volleyball players (mean age 28.32 ± 4.01) who voluntarily agreed to participate in the study. The anthropometric characteristics and the physiological parameters of the participants are presented in Table 1. All the participants were in their resting time after a sports competition and they were asked to avoid physical effort (except normal daily activity) during a whole month of resting. The first exercise of the experiment took place two weeks after the end of volleyball season, while the second exercise was performed two weeks after the first one. During the first stage of the study, prior to the exercise, the athletes were subjected to a single session of whole-body cryotherapy. Immediately after the exit from cryochamber (1-2 min), the participants were allowed up to 5 min to warm-up at an intensity of 1.5 W per kilogram of the body weight. Following the warm-up, the subjects began a 40 min submaximal exercise using a cycle ergometer Monark 828 E (Monark Exercise AB, Sweden). At the second stage of the study the sportsmen performed the same exercise but without any stimulation in the cryogenic chamber (control exercise). The frequency of heart contraction was the physiological criterion of the burdening of the organism. During both stages of the study, it was equal to ~85% HR_max⁡_ and was maintained during the whole exercise by all participants. The heart rate was monitored by means of a Polar H7 Heart Rate Sensor (Polar Electro Oy, Finland) connected to the PC. The mean power of the first exercise was about 165 W with pedaling cadence of about 50 rpm, while the second exercise was about 160 W with the same pedal frequency.

The systemic cryotherapy procedure was conducted by means of a single entry into the “Arctica” cryochamber which was manufactured by Metrum CryoFlex (Poland). This cryochamber is called an “entry-descent” cryochamber or a cryochamber with a cold deposit. The cryogenic chamber is chilled using synthetic vapors, liquid air, which is a homogenous mixture of nitrogen and oxygen (with 22% ± 2% of oxygen). The oxygen level in the cryotherapy chamber is controlled in two points and the monitoring sensor cuts off the power when the oxygen level is too high or too low. This increases the safety of the procedure. In order to make use of the “cold deposit” phenomenon, the cryogenic chamber was placed about 2.5 m under the floor level. The subjects enter the therapy chamber by stairs which constitute a mild adaptive area. An open vestibule, in which the temperature is kept at −60°C, is located at the base of the stairs. The vestibule and the main chamber are separated by double swing doors. During the procedure the temperature in chamber was maintained at −130°C. The temperature was controlled using a microprocessor controller operated from a PC computer. Temperature in such a chamber is dependant on the automatically regulated amount of liquid air injected into and evaporated out of the chamber. The “entry-descent” cryochamber is based on a wooden framework with the walls filled with a material with unique isolation qualities. The inner part of the chamber is lined with impregnated wood. A transparent ceiling enables keeping eye contact with the cryochamber crew and the lighting of the chamber with a phototherapeutic lamp increases the comfort and safety of the procedure.

Before entering the cryochamber, the subjects put on a safety suit which consisted of cotton shorts, acrylic socks and gloves, and band for auricle protection. Clogs were an additional mean of protection against feet frostbite. Dust masks were used for air passages protection. The soft outer layer of the mask which is supposed to stop dust constituted an outer filtration layer protecting from early ice dust clogging. Every subject was informed about the rules: the need for slow, shallow breathing (short nasal inhalation and longer oral exhalation), and the way to move about (slow walking in circles).

The total time a subject stayed in the cryochamber was not longer than 2 minutes. Every entry into the main chamber was preceded by 10–20 seconds of adaptation in the open vestibule. The sportsmen were qualified for the WBC based on a positive assessment of their health during a medical checkup. The research was realized with the consent of the Bioethics Committee at Collegium Medicum in Bydgoszcz of the Nicolaus Copernicus University in Toruń, Poland. All examined sportsmen provided written informed consent.

At the first stage of the study, the blood samples were obtained three times: before entering the cryochamber, right after the cryotherapy (before the exercise), and immediately after submaximal exercise on the cycloergometer. During the control stage, the blood samples were taken twice—before and after physical effort. The investigated parameters were determined using blood taken from a basilic vein. Venous blood was taken into two test tubes: one containing an anticoagulant, dipotassium tartrate (K_2_EDTA—ethylenediaminetetraacetic acid dipotassium salt), in order to obtain whole blood and the second was dry in order to obtain blood serum. The concentration of thiobarbituric acid reactive substances (TBARS) was determined in blood plasma and erythrocytes, whereas the level of conjugated dienes (CD), in the blood plasma of sportsmen. The activity of SOD, GPx, and CAT was assayed in the erythrocytes of the participants. Total oxidative status and the levels of selected cytokines, IL-1*β*, IL-6, TNF-*α*, and TGF-*β*1, were measured in blood serum.

### 2.1. Assay of Lipid Peroxidation Products in Blood Plasma and Erythrocytes

TBARS concentration was measured by means of Buege and Aust method [24] which was modified by Esterbauer and Cheeseman [25]. The products of lipid peroxidation were assayed with the use of thiobarbituric acid (TBA). The main product of lipid peroxidation reacting upon TBA is malondialdehyde (MDA), so for simplification the level of thiobarbituric acid reactive substances was expressed as concentration of MDA. The volume of 0.5 mL haemolysate or 0.5 mL blood plasma was added to 4.5 mL of the reaction mixture containing 0.375% TBA and 15% of trichloroacetic acid (TCA) in 0.25 M HCl. In order to prevent the formation of lipid peroxidation products during the reaction itself, 0.01% of the solution of butyl-4-hydroxytoluene (BHT) was added to the samples to cease the peroxidation process. The samples were incubated for 20 minutes in the temperature of 100°C. After cooling, they were centrifuged for 15 minutes at 2000 g in the temperature of 4°C. The supernatant extinction was measured at the wavelength of 532 nm. The concentration of TBARS in erythrocytes was expressed in nmol of MDA/g Hb, while that in blood plasma was expressed in nmol of MDA/mL of plasma. The intraassay and the inter-assay controls imprecision, as CV%, was 6.5–10.2% and 8.6–11.9%, respectively.

The level of conjugated dienes was determined according to Sergent et al. [26]. CD are formed during the process of lipid peroxidation, as the result of rearrangement of double bonds after the detachment of hydrogen atom from the rest of polyunsaturated fatty acid. They give a characteristic absorption peak at the wavelength of 233 nm. In order to determine the CD level, 0.5 mL of chloroform was added to 0.5 mL of the plasma, and then it was centrifuged and 0.1 mL of the solution was taken into clean test tube. The samples were vaporized in the nitrogen atmosphere, dissolved in cyclohexane, and then the absorbance readings at the wavelength of *λ* = 233 nm were performed. The conjugated dienes level was expressed in the absorbency units per mL of plasma (abs./mL). The sensitivity of this assay is up to a few nanomoles (2-3 nmoles). The assays had a within-run CV range from 5.7 to 7.3% and a between-run CV from 4.6 to 8.3%.

### 2.2. Assay of Antioxidant Enzymes Activity in Erythrocytes

Superoxide dismutase activity in erythrocyte haemolysates was evaluated in accordance with the method based on the enzyme impeding the reaction of auto-oxidation of adrenaline to adrenochrome in an alkaline medium [27]. The unit of activity of SOD is the quantity of the enzyme that impedes the reaction by 50% at a maximum increase in absorption of 0.025 units/min on a rectilinear section of adrenochrome formation. Glutathione peroxidase activity was measured in erythrocytes by detecting the changes in absorption caused by the change of the reduced form of nicotinamide adenine dinucleotide phosphate (NADPH) into an oxidized form [28]. NADPH is a coenzyme in the reaction of the reduction of glutathione disulphide catalyzed by glutathione reductase. The obtained oxidized glutathione is a product of the reaction catalyzed by GPx. The substrate was hydrogen peroxide. The activity of SOD and GPx is expressed in U/gHb. The Beers and Sizer method [29] was used to determine catalase activity. This method is based on the measurement of the absorbance decrease of hydrogen peroxide which is decomposed by catalase, measured at a wavelength of 240 nm. CAT activity is expressed in IU/gHb.

The optical density of samples was measured in the “Cary 100” spectrophotometer of Varian company (USA). The reagents were obtained from Sigma Company (Sigma-Aldrich LLC) and also from Polish Reagents Chemical Enterprise SA (Gliwice, Poland). The analytical performance of used methods is satisfactory with the intra-assay coefficient of variation (CV) between 6.4 and 13.6% and the inter-assay CV between 3.4 and 11.8%.

### 2.3. Assay of Total Oxidative Status and Cytokines Level in Blood Serum

The total lipid peroxides as the total oxidative status (TOS) in the blood serum of participants were determined using enzyme linked immunosorbent assay (ELISA) according to instructions given by the producer. For TOS determination, the commercial kits were used (Biomedica Company, Piaseczno, Poland). Total oxidative status was given in *μ*mol/L. The detection limit for the TOS tests was 7 *μ*mol/L.

In blood serum, the level of cytokines, IL-1*β*, IL-6, TNF-*α*, and TGF-*β*1, was also determined with use of a solid-phase ELISA kits, based on the sandwich principle. The measurements were performed in accordance with instruction given by the producer (Biomedica Company, Piaseczno, Poland). The principle of the method is based on binding of cytokines by monoclonal antibodies specific for given cytokine. An antibody was absorbed into microwells. During first incubation, cytokine present in the sample bounds to the antibodies adsorbed into the microwells. After washing a biotin-conjugated monoclonal, antibody specific for cytokine was added. During second incubation, this antibody bounds to immobilized cytokine captured during first incubation (by the first antibody). Following incubation, unbound biotin conjugated antibody was removed during a wash step with use of microplate washer (Thermo Fisher Scientific Oy, Finland). After removal of second antibody, streptavidin protein conjugated to horseradish peroxidase (streptavidin-HRP) was added and bound to the biotin-conjugated antibody to complete the four-member sandwich. After a third incubation and washing to remove all the unbound enzymes, a substrate solution reactive with HRP was added to the wells, which was acted upon by the bound enzyme to produce color. A colored complex was formed in proportion to the amount of cytokine present in sample or standard. The reaction was terminated by addition of acid and absorbance at *λ* = 450 nm was measured. A standard curve from standard dilutions and cytokines' samples concentration was determined. The intensity of colored product is directly proportional to the concentration of cytokine present in the original specimen. The level of IL-1*β*, IL-6, and TNF-*α* was expressed in pg/mL, while the level of TGF-*β*1 was expressed in ng/mL. The detection limits for cytokines tests were for IL-1*β*—0.32 pg/mL, for IL-6—0.92 pg/mL, and for TNF-*α*—4 pg/mL while being for TGF-*β*1—0.02 ng/mL.

### 2.4. Estimation of Physical Endurance Parameters of Participants

The estimation of maximal heart rate (HR_max⁡_) was measured by direct test on a cycle-ergometer Monark 828 E (Monark Exercise AB, Sweden). All participants performed a 5 min lasting maximal exercise, during which the heart rate was monitored by means of a Polar H7 Heart Rate Sensor (Polar Electro Oy, Finland) and the results were analyzed by PC software. VO_2_max was estimated by direct method in a stepped endurance test until exhaustion on a treadmill Cosmos Pulsar 3p4.0 (Cosmos Sports and Medical GmbH, Germany). During the measurements, the participants were connected to an ergospirometer START 2000 (MES Sp.z o.o., Poland) by Mask Hans Rudolph Series 8900 (Hans Rudolph Inc., USA). The measurement of aerobic capacity of the subjects was determined at the end of the volleyball season, two weeks prior to the start of the experiment.

### 2.5. Statistical Analysis

The results are expressed as means ± SD (standard deviations). Statistical analysis was performed by means of repeated measures ANOVA test with post hoc analysis (Tukey's range test) (*STATISTICA* v. 9.1). Before running ANVOVA with repeated measurements, model assumptions were tested using Kolmogrov-Smirnov test and the results met the normal distribution. Moreover, the homogeneity of covariance was confirmed by Levene's test. The dependencies between the analyzed parameters were estimated using correlation matrices. A statistical hypothesis of the significance of the correlation coefficient (*r*) was tested by means of Spearman's test. In all cases, the differences at the level of significance *P* < 0.05 were accepted as statistically significant.

## 3. Results

**Figure 1:**
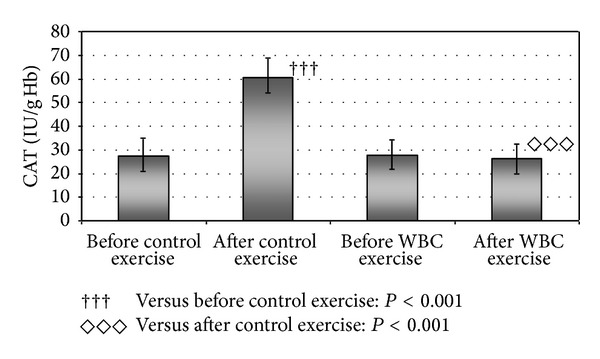
Statistically significant differences in the activity of catalase (CAT) in erythrocytes of volleyball players after exercise without whole-body cryostimulation (control exercise) and exercise preceded by whole-body cryostimulation (WBC exercise).

No statistically significant changes in the level of CD, TBARS, GPx, and TOS in the blood of volleyball players were found after cryotherapy or after exercise in either condition (Table 2). However, the activity of CAT in the subjects' erythrocytes after the control exercise was about two times higher (*P* < 0.001) than before the control exercise (Figure 1). Moreover, comparing the activity of CAT and SOD after WBC exercise and control exercise, some significant changes were observed. The activity of catalase in volleyball players' erythrocytes after WBC exercise was about two times lower (*P* < 0.001) when compared with the activity right after control exercise (Table 2). The activity of SOD in the sportsmen erythrocytes was also about 8% lower after WBC exercise than after control exercise (*P* < 0.05) (Table 2).

**Figure 2:**
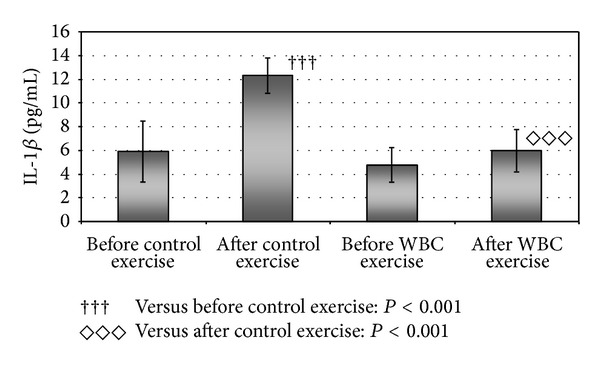
Statistically significant differences in the level of interleukin-1*β* (IL-1*β*) in blood serum of volleyball players after exercise without whole-body cryostimulation (control exercise) and exercise preceded by whole-body cryostimulation (WBC exercise).

**Figure 3:**
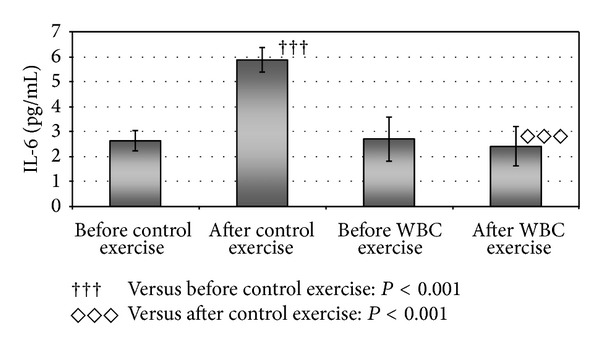
Statistically significant differences in the level of interleukin-6 (IL-6) in blood serum of volleyball players after exercise without whole-body cryostimulation (control exercise) and exercise preceded by whole-body cryostimulation (WBC exercise).

The level of IL-1*β* (Figure 2) and IL-6 (Figure 3) statistically significantly increased after control exercise and it was about two times higher than before control exercise (*P* < 0.001) (Table 3). There were no statistically significant changes in IL-1*β*, IL-6, TNF-*α*, and TGF-*β*1 level in blood serum of studied volleyball players after whole-body cryotherapy session and after WBC exercise (Table 3). However, comparing the exercise with and without systemic cryotherapy, statistically significant differences in IL-1*β* and IL-6 level were revealed (Table 3). The level of those cytokines was about twice lower after WBC exercise as compared to control exercise (*P* < 0.001). After WBC procedure, statistically insignificant deceasing tendency was observed in level of TNF-*α* (right after exposure to extremely low temperatures and after WBC exercise).

**Figure 4:**
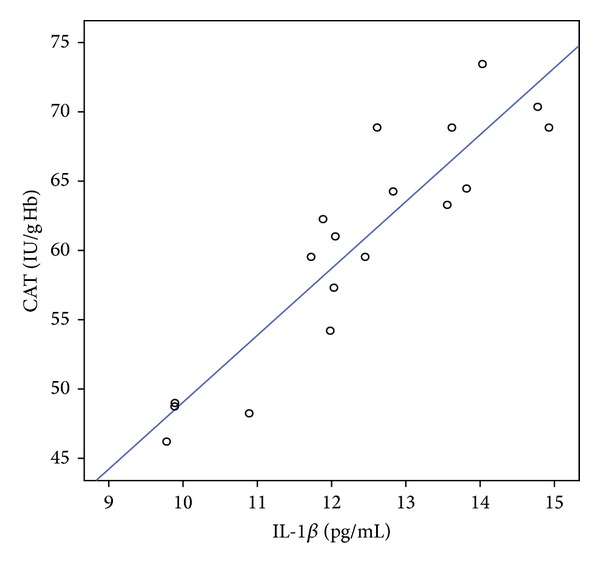
Linear regression (*r* = 0.912, *P* < 0.05) of catalase (CAT) activity versus interleukin-1*β* (IL-1*β*) level at blood of volleyball players after exercise not preceded by whole-body cryostimulation (control exercise).

In presented study, some relevant correlations between markers of oxidative stress and inflammation were also observed. After control exercise negative correlation (*r* = −0.829, *P* < 0.05) was found between IL-1*β* level in blood serum and TBARS level in blood plasma, while positive correlation (*r* = 0.912, *P* < 0.05) was found between IL-1*β* level in blood serum and CAT activity in erythrocytes (Figure 4). After single cryotherapy session, negative correlation between TNF-*α* level in blood serum and CD level in blood plasma was found (*r* = −0.978, *P* < 0.05) and between IL-6 level in blood serum and SOD activity (*r* = −0.798, *P* < 0.05) in erythrocytes. After WBC exercise, negative correlation was observed between IL-6 level and GPx activity (*r* = −0.884, *P* < 0.01), between level of IL-1*β* in blood serum and TBARS in erythrocytes (*r* = −0.926; *P* < 0.05), and between TGF-*β*1 level in blood serum and GPx activity in erythrocytes (*r* = −0.917, *P* < 0.05).

## 4. Discussion 

In this study, no statistically significant changes in the level of CD, TBARS, and total TOS in the blood of volleyball players after a 40 min submaximal control exercise were found. Those results are in agreement with the research of other authors who observed no changes in the level of lipid peroxidation products after the exercise [30–32]. There were also no statistically significant changes in the activity of antioxidant enzymes like SOD and GPx in the blood of volleyball players who underwent submaximal exercise. However, the activity of catalase significantly increased after control exercise. Postexercise increase of CAT activity was also observed by other authors [33–35]. The increase of antioxidant enzymes activity measured in cellular material is usually related to elevated enzyme synthesis. However, an increase of CAT activity in erythrocytes cannot be caused by *de novo *enzyme synthesis as these blood elements do not have a cell nucleus. Hence this increase is probably connected with the presence of agents which modify the speed of enzymatic reaction. Catalase destroys hydrogen peroxide (H_2_O_2_) by catalysing its two-electron dismutation into oxygen and water but only at high concentration of H_2_O_2_ [36]. Moreover, the rate of its activity is proportional to H_2_O_2_ concentration over a wide range of the latter and is among the highest enzymatic rates [37]. The increase in CAT activity proves that, in studied athletes, significant rise of hydrogen peroxide level occurs immediately following control exercise. Accumulating evidence suggests that ROS are not only injurious byproducts of cellular metabolism but also essential participants in cell signaling and regulation [38]. Among ROS, H_2_O_2_ seems to play the crucial role in this process, as unlike free radicals it is a stable molecule, which is able to diffuse across the biological membranes [39]. Hydrogen peroxide may contribute to regulation of gene expression by inactivation of membrane-bound protein tyrosine phosphatase or by modulation of redox-sensitive transcription factors like nuclear factor-kappa B (NF-*κ*B) and activator protein-1 (AP-1) [22, 40]. The genes induced by NF-*κ*B are well known to encode proteins involved in immune system response, like acute phase proteins and myokines [41].

In the presented paper, statistically significant increase of IL-1*β* and IL-6 level was observed in blood serum of studied subjects immediately after the exercise. Those results match with the finding of Laskowski et al. [42] who revealed increased level of IL-6, IL-1*β*, and TNF-*α* in the blood of athletes immediately after judo training and the strongest effect was observed in case of IL-1*β* level, which correlated with creatine kinase activity. Numerous studies indicate a substantial increase in the level of IL-1*β* after prolonged and intense exercise and its elevated level is usually due to microinjuries of muscle tissue [43]. The increase of IL-6 during exercise has been also welldocumented and this cytokine is an essential regulator of the proliferation of satellite cells and differentiation of myoblasts [44]. Zembron-Lacny et al. [45] observed increased level of IL-6 20 min after run at 65% VO_2_max and a strong positive correlation between this cytokine and hydrogen peroxide level. In our study, a strong positive correlation was found between catalase activity and IL-1*β* level in blood of participants, which implies that increased level of cytokines found in this study is probably due to high amount of H_2_O_2_ generated in muscle tissue during exercise. Febbraio and Pedersen [44] demonstrated that the main source of IL-6 in circulation in response to exercise is contracting skeletal muscle *per se*, not the immune cells. Furthermore, Kosmidou et al. [46] proved that expression of IL-6 gene is directly proportional to the concentration of hydrogen peroxide generated during oxygen metabolism.

Following the aim of the study, an attempt was made to determine the role of single whole-body cryotherapy applied prior to exercise in reduction of exercise-induced oxidative stress. In the presented paper, no statistically significant changes in the level of oxidative stress markers in blood of volleyball players were observed right after the exit from cryochamber. Yet, when comparing the exercise preceded and not preceded by WBC, the activity of CAT and SOD was significantly lower in blood of subjects after WBC exercise than after control exercise. Lubkowska et al. [47] also found significant decrease in the activity of catalase and glutathione transferase 30 min after single whole-body cryotherapy in the blood of healthy subjects. Similar results were demonstrated in male and female kayak athletes when cryogenic sessions were included into the training protocol [16, 48].

The results obtained in the presented paper are likely to be a consequence of a decreased ROS generation and/or significant participation of nonenzymatic systems in their removal due to cryogenic temperature action. Dugué et al. [49] demonstrated that during the first 4 weeks of WBC sessions, the mean value of total peroxyl radical trapping antioxidant capacity of plasma (TRAP) in healthy women significantly increased at 2 min after cryotherapy, returning to baseline 35 min after the cold exposure. The increase of total antioxidant status and the level of uric acid as a result of series of short-term whole-body cryotherapy was also observed by Miller et al. [50] in blood of healthy subjects. Lubkowska et al. [47] revealed the insignificantly increased level of uric acid 30 min after WBC and the significant increase of this antioxidant one day after cryotherapy. The lowered CAT activity observed in the presented paper may testify to the reduced level of hydrogen peroxide, when exercise is preceded by WBC. As it was previously explained, H_2_O_2_ participates in the expression of cytokines genes; thus the decreased level of this ROS may be reflected in the lowered level of cytokines released under the influence of exercise. Rhind et al. [51] observed increased expression of genes encoding IL-1*β*, IL-6, and TNF-*α* in young healthy volunteers immediately after the sports training, but when the training was preceded by exposure to low temperatures the expression of IL-1*β* and TNF-*α* was significantly lower. In the present study, we found that the level of IL-6 and IL-1*β* was about twice lower after WBC exercise as compared to control exercise, while TNF-*α* and TGF*β*-1 remained unchanged. Those findings are consistent with research of Pournot et al. [3], who observed that level of IL-1*β* in blood of athletes subjected to intense simulated trail run was lower, when WBC procedure was included to recovery sessions following the exercise. The changes in cytokines levels (decreased TNF-*α* and increased IL-6) were also observed in professional tennis players and the authors believe that WBC, which shows anti-inflammatory effect, may function as a support for training program to ensure an effective midseason break [52]. In present paper, no statistically significant changes were found in the level of studied cytokines in the blood of athletes immediately after a single cryotherapy and similar results were obtained by Leppäluoto et al. [11]. Lubkowska et al. [53] reported the increased level of IL-6 accompanied by the increase of IL-10, without changes in IL-1*α*, IL-1*β*, and TNF*α* after only five daily cryostimulation sessions. The increase of IL-10 level with concomitant decrease of IL-2 and IL-8 levels in the blood of professional rugby players after a series of 5 WBC interventions during one-week regular training was also demonstrated by Banfi et al. [6]. Presented reports on the effect of cryogenic temperatures on the cytokines levels are contradictory making hard to formulate unambiguous conclusion, mainly due to the differing WBC procedures in above-presented experiments. The WBC interventions differed in number of treatments and duration of single exposure. Moreover, WBC was used in the course of training cycle in the athletes as well as in healthy, untrained subjects.

The lower activity of CAT and SOD accompanied by lower level IL-1*β* and IL-6 after WBC exercise may result from the activation of heat shock proteins (HSPs), which have been proposed to play a direct role in protecting against damage caused by ROS [54, 55]. An increase in the level of HSPs in response to cold stress was observed in rats [56] and in human fibroblast cell cultures [57], but the research on the effect of WBC on HSPs level is sparse and it may be an interesting direction for future research. Ziemann et al. [58] demonstrated that HSP27 negatively correlated with H_2_O_2_ and proinflammatory cytokines levels in young tennis players during a camp aiming to reduce overreaching syndrome.

The obtained results confirm the anti-inflammatory action of single WBC intervention prior to exercise and its beneficial role in reduction of the signs of exercise-induced oxidative stress in the athletes. Yet, it is still unclear whether the decreased postexercise CAT activity is due to reduced H_2_O_2_ production after WBC exercise and its consequence is the lowered level of cytokines or maybe the single WBC procedure reduces the release of cytokines, which results in limited ROS production by immune cells reflected by decreased CAT activity. Few studies suggest that the immunomodulatory effect of cryotherapy observed in patients with various diseases is caused by changes in the quantity of certain cytokines [59]. Limited evidence suggests that cold exposure may initiate changes in cytokine expression associated with a nonspecific acute-phase reaction related to interactions between the cytokines and neuroendocrine hormones [60]. Moreover, cold may inhibit the expression of inflammatory mediators, which normally act as potent vasodilators and cause an increase in vascular permeability and edema. Banfi et al. [6] found that cold treatment induced the decrease of adhesion molecule-1 (sICAM-1) correlated to decrease of prostaglandin E2 (PGE2), which is a central mediator of febrile response triggered by inflammatory process. The observed changes in inflammatory response to physical activity as a result of WBC action are probably also associated with cold-induced vasoconstriction related to reflex sympathetic activity and attendant increase in the affinity of adrenoreceptors for norepinephrine. Stimulation of *β*_2_-adrenoreceptor is known to be manifested by a decrease in the synthesis of proinflammatory cytokines with concomitant stimulation of anti-inflammatory cytokine production [61].

## 5. Conclusions

Obtained results suggest that even a single application of cryotherapy prior to exercise may have a beneficial impact on antioxidant system of organism and alleviate the signs of exercise-induced oxidative stress. The differences in the profile of cytokines after control exercise and WBC exercise imply that exposure to extremely low temperatures may regulate the inflammatory response of organism to physical effort, although the cellular mechanism of the observed changes is still unclear and requires some further studies. As observed in this study the correlations between the markers of oxidative stress and inflammation may testify to the important contribution of ROS in inflammatory response to physical exercise.

## Figures and Tables

**Table 1:** Physical characteristics of the studied group.

Parameter	Volleyball players
Number of subjects	18
Age (years)	28.32 ± 4.01
Body mass (kg)	87.1 ± 7.36
Body height (cm)	192 ± 9.12
BMI (kg/m^2^)	23.63 ± 1.12
HR_max_ (beats/min)	186 ± 4.78
VO_2_max (mL/min/kg)	61 ± 2.28
Training period (years)	11.8 ± 3.2

Values are expressed as means ± standard deviations (SD) of the means.

**Table 2:** The concentration of lipid peroxidation products: thiobarbituric acid reactive substances (TBARS) and conjugated dienes (CD), the activity of antioxidant enzymes: catalase (CAT), glutathione peroxidase (GPx), and superoxide dismutase (SOD), as well as serum total oxidant status (TOS) in blood of volleyball players before and after physical effort preceded by whole-body cryotherapy (WBC exercise) and exercise without stimulation by cryogenic temperatures (control exercise).

	WBC exercise			Control exercise	
	Before WBC and exercise	After WBC (before exercise)	After exercise preceded by WBC	Before exercise	After exercise
TBARS_plasma_ (10^−2^ nmolMDA/mL)	60.3 ± 10.9	59.4 ± 7.8	59.1 ± 9.5	51.8 ± 11.3	57.6 ± 5.13
TBARS_erythrocytes_ (nmolMDA/gHb)	44.6 ± 15.2	48.8 ± 14.5	52.4 ± 11.4	48.1 ± 14.3	51.6 ± 16.2
CD_plasma_ (10^−2^ Abs./ML)	11.5 ± 2.4	10.9 ± 3.6	12.3 ± 2.7	9.85 ± 5.7	10.8 ± 3.4
SOD (U/gHb)	1523.2 ± 167.7	1576.4 ± 254.3	1489.4 ± 113.4	1587.7 ± 234.5	1598.7 ± 129.6*
GPx (U/gHb)	4.71 ± 1.1	5.52 ± 2.6	5.03 ± 1.3	4.88 ± 1.9	4.92 ± 2.4
CAT (10^4^ IU/gHb)	27.82 ± 6.5	29.71 ± 4.1	26.24 ± 6.3	27.21 ± 7.8	60.52 ± 8.3^∗∗∗aaabbbccc^
Serum TOS (*µ*mol/L)	177.2 ± 65.5	168.5 ± 63.6	192.5 ± 48.3	198.4 ± 75.2	212.6 ± 66.4

Values are expressed as means ± standard deviations (SD) of the means. Statistically significant differences: versus after exercise preceded by whole-body cryotherapy: *P < 0.05; ***P < 0.001; versus before WBC and exercise: ^aaa^ *P* < 0.001; versus after WBC: ^bbb^ *P* < 0.001; versus before control exercise: ^ccc^ *P* < 0.001.

**Table 3:** The level of selected cytokines: interleukin-1*β* (IL-1*β*), interleukin-6 (IL-6), tumor necrosis factor *α* (TNF-*α*), and transforming growth factor *β*1 (TGF-*β*1) in blood serum of volleyball players before and after physical effort preceded by whole-body cryotherapy (WBC exercise) and exercise without stimulation by cryogenic temperatures (control exercise).

	WBC exercise			Control exercise	
	Before WBC and exercise	After WBC (before exercise)	After exercise preceded by WBC	Before exercise	After exercise
IL-1*β* (pg/ML)	4.78 ± 1.5	4.59 ± 2.4	5.96 ± 1.8	5.93 ± 2.6	12.34 ± 1.5^∗∗∗aaabbbccc^
IL-6 (pg/ML)	2.72 ± 0.9	2.81 ± 0.4	2.42 ± 0.8	2.63 ± 0.4	5.88 ± 0.5^∗∗∗aaabbbccc^
TNF-*α* (pg/ML)	7.61 ± 2.5	5.72 ± 1.3	4.91 ± 1.4	8.22 ± 2.7	7.71 ± 1.6
TGF-*β*1 (ng/Ml)	34.8 ± 8.4	37.9 ± 8.8	38.6 ± 7.6	38.2 ± 9.7	38.7 ± 6.7

Values are expressed as means ± standard deviations (SD) of the means. Statistically significant differences: versus after exercise preceded by whole-body cryotherapy: ***P < 0.001; versus before WBC and exercise: ^aaa^ *P* < 0.001; versus after WBC: ^bbb^ *P* < 0.001; versus before control exercise: ^ccc^ *P* < 0.001.
